# Brazilian Specialists’ Perspectives on the Patient Referral Process

**DOI:** 10.3390/healthcare5010004

**Published:** 2017-01-29

**Authors:** Carmen Juliani, Maura MacPhee, Wilza Spiri

**Affiliations:** 1 Department of Nursing, São Paulo State University UNESP, Botucatu 18618-970, Brazil; wilza@fmb.unesp.br; 2 School of Nursing, The University of British Columbia, Vancouver, BC V6T 2B5, Canada; Maura.MacPhee@nursing.ubc.ca

**Keywords:** referral, consultation, specialties, primary care, communication, phenomenology

## Abstract

Since 1988, healthcare has been considered a citizen’s right in Brazil. The Sistema Único de Saúde (SUS), has undergone development and expansion to ensure universal health coverage for the Brazilian public, the world’s fifth largest population. The coordination of effective communications between primary care physicians, specialists and patients is a significant challenge, particularly the referral process. Our study objective was to understand the facilitators and barriers associated with referral process communications between primary care physicians and regional university hospital specialists in the State of Sao Paulo. This paper reports specialists’ perspectives of the referral process. This was a phenomenological study that employed a qualitative research method with three components (description, reduction and comprehension). We conducted focus groups with 54 hospital residents from different specialties (surgery, medicine, obstetrics/gynecology, pediatrics) from July to October 2014. The main results showed lack of an adequate referral-return referral process resulting in treatment delays and inappropriate use of emergency services. Communications were impeded by lack of integrated, computerized booking and standardized referral-return referral processes; underlying lack of trust in primary care physicians; and patients’ inappropriate use of healthcare services. Although computerized systems will facilitate communications between primary and specialty care, other strategies are needed to promote collaboration between services, and ensure appropriate utilization of them.

## 1. Introduction

Within Brazil, a national priority has been the establishment of an integrated health care network that provides basic, universal health coverage for a population with burgeoning health care needs associated with non-communicable and chronic diseases. The Brazilian public healthcare system, Sistema Único de Saúde (SUS), was designed to include all citizens. Health is considered a citizen’s right and duty of the state [1,2,3]. Integrated health care networks or systems are considered more effective and efficient means for linking patients to services. Links within and across different services or systems levels (e.g., primary care, secondary and tertiary care) require systematic information and knowledge management [4]. Systems segmentation and fragmentation are common problems in low and middle-income countries, such as Brazil [5]. Our study explored specialist physicians’ perspectives on one critical link, the referral process, between community-based primary care services and specialists within tertiary care hospital settings.

Primary care services focus on families and communities and emphasize health promotion and public health initiatives (e.g., immunization). Primary care family clinics in Brazil are staffed by teams comprised of a doctor, one to two nurses and four to six community health workers. They are assigned to specific geographical areas with 600–1000 families [6].

### Background

In Brazil, there are rising demands for primary and specialized care. The integration and coordination of communications between different health system levels requires an effective referral process between primary care physicians and specialists.

The referral process (referral/return referral) is initiated when a patient’s primary care physician encounters a complex medical situation that requires a specialist consultation. As ideally planned, the referral request to a specialist provides basic patient information: the return referral to the primary care physician includes the specialist’s thorough diagnostic evaluation and a treatment plan [7]. Our previous research examined primary care physicians’ referral processes [8,9]. Primary care physicians reported barriers similar to findings from other countries. They were particularly hampered by heavy workloads and patients requiring chronic disease management. In Brazil, for instance, 50% of the public over 40 years of age is hypertensive, and 6 million have diabetes [8]. Notably, primary care physicians complained about the lack of return referrals from specialist consultants, or returned referrals with no helpful diagnostics or recommendations. This study explores the referral process from specialists’ perspectives; to help us, as researchers, determine the facilitators and barriers to requisite knowledge sharing between services.

The timing of this study was important due to the anticipated transition from a paper and pencil referral process to an electronic medical record (EMR) process. Prior to this technology transformation, it is critical to examine the nature of communications between primary care physicians and specialists; to ensure that relational aspects of the referral process are operating effectively between different systems levels before the introduction of EMR. In addition, the Brazilian government is making significant investments in primary care delivery; shifting care from the acute care sector to the community. This planned care delivery transition will be enhanced by smooth communications links between physicians in the community and necessary specialist supports [6]. One recent mixed methods study within the Veteran Hospital Administration (VHA) found that a shared EMR was no panacea for an effective referral process. Effective referral communications were influenced by opportunities for primary care-specialist physicians to engage in other forms of dialogue and more personal interactions (e.g., telephone, face-to-face) that promoted collaborative relationships and trust among physicians [10].

A number of studies have shown how ineffective referral communications can undermine planned shifts from hospital-based care delivery to community-based primary care delivery. In the U.S., for instance, one national health goal has been to employ a primary care model, the patient-centered medical home (PCMH), as a major form for healthcare delivery [11]. Patients are being encouraged to obtain the majority of their care from PCMHs. National trends, however, are showing that referrals to specialty services and use of these more expensive services are not decreasing. One rationale is lack of effective communications between primary care physicians and specialists, resulting in unnecessary referrals for specialty care [10].

Other research has studied those environmental factors associated with referral communications. Scarcity of resources, including insufficient numbers of community-based physicians and lack of technology, contribute to ineffective referral communications [12]. Initiating and sustaining consistent care coordination of patients between services is threatened by lack of care provider continuity. Environments with high physician turnover, therefore, are at risk for referral communications breakdowns. Layman and Bamberg [13] defined environmental complexity as the numbers and types of environmental factors that physicians must consider when making patient care decisions. They found that when physicians had to juggle multiple demands, they became more indecisive and less confident in their own decisions. A cross-sectional study with American Medical Association data found that patient complexity (e.g., poorer health status, co-morbidities) negatively influenced referral communications. The researchers surmised that complex patients increase physicians’ workloads, decreasing their opportunities to provide in-depth information during primary care-specialist communications [14].

As in other parts of the world, improving the overall referral process in Brazil necessitates further examination of the communications between providers. Our research question, grounded in our previous study was: What are the barriers and facilitators to the Brazilian referral process from the specialists’ perspectives?

## 2. Materials and Methods

Qualitative research with Phenomenology builds knowledge by exploring people’s daily life experiences [15]. We used a specific phenomenological approach based on the work of Merleau-Ponty [16] and adapted by a Brazilian phenomenologist, Martins [17]. Martins’ method has three research components with eight procedural steps: (a) *description* (obtain data, read/reread the statements in full); (b) *reduction* (identify the “unit of meaning” or essence of speech, reduce or eliminate unnecessary words); and (c) *comprehension* (interpret and organize the “units of meaning” into themes and sub-themes, connect themes and sub-themes to the literature).

### 2.1. Population and Sample

A convenience sample of focus group volunteers was recruited from a population of 92 physician residents in specialties at the regional hospital in the State of Sao Paulo, Brazil. In Brazil, all physicians begin their training in Health Basic Units (HBUs) or primary care clinics. They then proceed to specialist residencies, managing the major volume of patients in specialty care. One regional hospital is a referral center for 68 municipalities. Fifty-four residents from different specialties (pediatrics, surgery, medical clinic, gynecology and obstetrics) in this hospital participated in five focus groups organized by specialty area. These specialties are most frequently represented in the referral process [9].

### 2.2. Data Collection

Each specialty residency group is under the direction of a chief resident. Study advertisements were circulated via the chief residents, and convenient times for focus groups were arranged with chief resident consultation. Potential participants were asked to contact the lead researcher via e-mail. Cover letters, consents and focus group information were sent to potential participants via e-mail, and a reminder e-mail was sent out 48 h before focus group sessions. Before each focus group session, time was allotted for questions and signed consents were collected.

Four semi-structured topics were presented to each focus group for discussion. The topics were constituted from previous findings [8]: (a) The importance of specialist return referrals for excellent patient care; (b) barriers or issues related to the referral process from a specialist/consultant perspective; (c) the overall effectiveness of the referral process within the SUS; and (d) recommendations for referral process/communications improvement. Questions or prompts were used to facilitate discussion around each topic area. Focus groups were held in private, quiet locations within the hospital from July to October 2014. Sessions were audio-recorded and lasted a maximum of 50 min.

### 2.3. Data Analysis

The recordings from focus groups were professionally transcribed from Portuguese to English. English transcriptions were coded by an English-speaking researcher, and Portuguese transcriptions were coded by a Portuguese-speaking researcher. For comparison purposes, Portuguese sub-themes and themes were translated into English. After independent coding, the researchers compared sub-themes and themes for consensus and selected exemplar quotes for sub-themes/themes. Coding and sub-theme/theme comparisons were done using NVivo9^®^ software QRS International (QSR International Pty Ltd., Doncaster, Australia). The eight procedural steps of Martins’ phenomenological methods were followed by both researchers.

### 2.4. Ethical Considerations

The study received institutional permission from the Research Ethics Committee of Botucatu Medical School (São Paulo State University) under the governance of the Brazilian National Council of Research with Human Beings (number 689.634/2014).

## 3. Results

### 3.1. Participants Profile

We asked focus group participants to provide basic demographics data. As noted in Table 1, our sample was over half of this regional hospital’s specialist residency population. Across the specialties, the average age of participants ranged from 25.6 to 28 years of age. The proportion of males and female participants varied by specialty, but for every service except surgery, there were greater proportions of female to male residents: This pattern is characteristic of the total residency population for this hospital.

### 3.2. Phenomenological Analysis

Inter-rater agreement between the Portuguese-speaking and the English-speaking researchers was reached in the coding process. Differences in coding were resolved through transcription review and discussion with a third researcher, fluent in English and Portuguese. Our analysis produced four themes with eleven sub-themes and 30 categories. The categories reflect the actual words (“meaning units”) from the transcripts. Table 2 is a summary of the coding schema that resulted from this analysis. Exemplar quotes are provided for sub-themes and categories. The themes are bold and sub-themes and categories are italicized.

**Theme: Government/Administrative Accountability.** This theme captures participants’ discussion of government/administrative accountability with respect to SUS public healthcare delivery. The major sub-themes were: physician workloads, physician turnover, the referral process (between HBU and specialties), and resources to ensure SUS maintenance and sustainability. *Physician Workloads*: “You have to set priorities in our minds—and the return referral is often not a priority. Now with the *number of patients* you have, some with *urgent needs.*” “The main thing is *time.* There is no way to stop the patients care for referrals report.” “The demand is very high, if you are thinking about stopping to fill in papers—we already have too much *bureaucracy*: two copies of prescriptions, and each exam you order has to have a separate form. It disrupts service. Doctors choose to treat patients instead of giving priority to the bureaucracy. I believe it’s correct to see patients and not papers.” Thus, specialist physician workload is a challenge because specialists give priority to patient care over writing return referral reports.

*Physician Turnover (in the HBU)*: From the specialists’ perspective, patients often bypass the HBUs, showing up at acute care centres for care due to their lack of stable relationships with primary care physicians. “Doctors don’t seem to stay more than one year in the HBUs. Doctors start out there with less experience—their goal is to move on because there is *no career* in HBUs, wages are low, and therefore, there is no continuity in primary care services.” “It’s rare for patients to have the same doctors for long in the HBU. Without the bond or link between physicians and patients, patients have no incentive to go to the HBUs. This is unrewarding to physicians as well. I think *stable doctor-patient relationships* in the HBU will encourage HBU use *Referral Process*: The referral process is hampered by a number of systems issues. “We have a long waiting list in Gyn (gynecology) and we have to organize the waiting list because we have *no trained administrative support* or staff to help us.” “Clinic management is quite complicated because there is *no logical organization of the patient list*. In most cases, the list is based on order of arrival—rather than complexity and need. I think this is because most of the administrative help are not healthcare professionals and they are not trained.” “We become so over-crowded, because there is *no triage or screening* beforehand. If patients could be screened earlier before ending up here, care would be better…many patients come to see us who should not be here.” “Things work well in our specialty clinic because we use a lot of *standardized guidelines and protocols*. For elective surgeries, for example, we have routines and protocols to support outpatient surgery scheduling. Things break down without them.” “I’ve worked at HBU and I thought, when I was at the HBU, that the specialist didn’t want to do the return referral. But the problem is there is *no direct link* between the doctor and the specialist. The general practitioner at HBU makes a written document that goes via the patient. Many times this referral does not reach the specialist. And similarly, the return referral never reaches the general practitioner. There is no link between care providers.”

*Resources*: Similarly, specialists identified specific resources that negatively influence the referral process. “I believe that there is a *lack of infrastructure in primary health care.* Even if the physicians in the HBU have good will and the intellectual capacity to manage patients in the community, there is insufficient infrastructure to do so. I recently saw a patient with tuberculosis in the infectious diseases specialty. Despite my assessment and instructions, I know that this patient will lack adequate monitoring in the community.” “We received a referral, a request for an evaluation. But we ended up sending this patient to the hospital for care. We did this because this patient came from a municipality without the means to manage this patient’s need for antibiotics.”

**Theme: Physician Accountability.** This theme captures specialist perspectives of problems with professional accountability among primary care physicians and among specialists.

*HBU Physicians**:*** “Sometimes, simple things that could be treated in the HBU end up being forwarded to specialty—before trying a treatment... I think this is due to physician *lack of education*—to know the right role and level of care.” “I think the HBU referral process is like ‘*forwarding therapy’*... The doctor acts like *‘passing the ball’* especially in pediatrics, where most of the HBU doctors are not pediatricians, and they send us patients because they can’t deal with it. Once I saw a physician do a hematology referral for typical iron deficiency anemia. He didn’t know how to interpret the exam and labs.” “The *HBU rarely calls* asking about patients-even when someone is hospitalized by us to find out how things are going.” “Wow, I’ve never received a call from the HBU.” “It’s rare to get follow-up after discharge, after a consultation.” “The form for referrals and return referrals is good, the problem is who fills it…and who doesn’t fill it out. ...or how they fill it out. There is unreadable handwriting, missing information, no diagnostic hypotheses or explanations.” “I’ll get referrals and not know what the primary wants.” “Sometimes it seems like *professional failure*—we end up doing a consultation without knowing what the patient really has.”

The quotes of specialists demonstrate their rationale for decreased feedback (i.e., return referrals), particularly their lack of confidence in primary care physicians. These quotes also highlight breakdowns in communications between primary care and specialist services. 

*Specialists:* “I see that there *is large distrust* between tertiary care and primary care. I am confident of care I gave in the HBU, but I’m not confident of the care of all my colleagues in the HBU. I see how many patients are referred in the wrong way to tertiary services.” “Sometimes there is a *bit of prejudice* ‘Oh, the doctor of the HBU will not know how to do this.’ ‘Oh, there is no need to do it, he will not know how to do, he will not understand.’ “We do it among ourselves too-sometimes we don’t understand the need for internal referrals when a patient is sent from one specialty to another. It is only written: ‘referral to this specialty.’ It is not just a matter with the HBU. Even among our own specialties, we *lack the conversation and the consideration* of how to do referrals well.” Professional trust and accountability, therefore is a problem for specialists with respect to HBU physicians and among themselves-specialist-to-specialist. 

**Theme: Patient Accountability or Vulnerability?** This theme reflects specialists’ discussions of whether patients are accountable for their behavior-or whether they are vulnerable “third parties” in a public healthcare system with many flaws.

*Patient Vulnerability*: “Many people only have contact with healthcare in emergency or acute situations—we send them for services that they do not understand. They are *naive*—they don’t know any better.” “Sometimes patients come by bus from small cities. They have *no place to go*…Sometimes they’re simple people who don’t understand the system. Sometimes the patients sit in wait in the ER of the hospital. Every day this happens…we see people, ‘spawn of this problem,’ that the city can’t handle.” “There is a *fragile third-party* in this relationship. The patient is expected to be the connection between services.”

*Patient Accountability*: “The patient already comes with the idea that the ER is faster than the HBU. *Primary care is devalued*. “It’s *cultural*… the patient says: ‘I have a fungal infection in the foot and my doctor can’t solve it. I want a dermatologist.” “They say: ‘Nothing gets solved in the HBU.’ They come to the ER or they want specialist referrals because they think specialty services will order lots of exams. They love CTs, MRIs and want to do a lot of exams.”

**Theme: Specialist Recommendations**. This theme encapsulates specialists’ recommendations for systems improvements. Recommendations are organized under three sub-themes: government/administration recommendations, physician recommendations and patient/public recommendations.

*Government/Administration*: “So I think we should have *more education for the population* related to the functioning of the health system in Brazil. The government should launch brief videos explaining how the HBU can help them with things like chronic headache—and when they should seek emergency help, like acute chest pain.” “*Education* of professionals too (about the SUS) and how to properly complete documentation.” “I think an ideal situation would be *computerized patient records available throughout the whole country*. That would be utopia.” I think, for the whole country, we should have *computerized forms* and a *referral system that is integrated across services*, like the HBUs, the ER, specialty clinics. I don’t think it is impossible for us to have access to critical patient information, no matter where we practice.” “Cases that should be solved in the HBU are not managed there because *there are not enough doctors*, or doctors with certain skills, such as obstetricians.” “I was in the ER today, and I got 5 cases from an HBU because the prenatal doctor did not show up. Then nurse who was there said: ‘Go to the hospital-the doctor will see you.”

*Physicians**:*** “I suggest that we need a culture, a sense of *professional accountability* to follow a systematic referral process between the HBU and specialties. We need to avoid the forwarding therapy approach (from HBUs) and the assumption that return referrals (from specialties) don’t matter. We need to both properly follow the patient.” “Our communications reflects our service to patients. We should establish a system for *communicating regularly* between us. To be able to talk on the phone to ask questions and give answers… to clarify doubts. We’ve kind of said it that communication is flawed.”

*Public/patients*: “When I was in the ER, if the patient came with a complaint that was taking too long, I would contact an *Ombuds service* to help.” “*Education of the population* is critical. “The public does not know how health services work. They do not understand that the HBU is the gatekeeper.”

## 4. Discussion

Starting with the first theme, **Government/Administrative Accountability**, the specialists in this study had been in HBU roles: their knowledge of HBU structures and processes helped them recognize challenges associated with primary care delivery and the referral process. They described their own workload challenges, and they recognized heavy workload demands on HBU physicians. In addition, they cited HBU physician turnover as a significant problem in the SUS-a source of unstable physician-patient relationships with negative impact on physician confidence and patient trust.

Primary care is considered the cornerstone of integrated health systems delivery, necessitating an investment in primary care physicians who should be the first point of health promotion/disease prevention within community settings [18].

The participants noted the inequity of resource distribution between primary care and tertiary care and recommended government intervention to improve HBU care delivery. Participants said that their return referrals were influenced by lack of resources for HBUs. In fact, in many instances, participants indicated that return referrals weren’t made to HBUs because these services lacked the sophistication and/or the means to provide follow-up care. For these patients, care was provided in the hospital, taking up valuable bed space. Systemic issues, such as resource distribution, therefore, influence provider communications across services. Our study participants recognized the importance of standardization-for referral forms and process-across the SUS. They also discussed the slow conversion of the SUS to an integrated EMR, partially due to inequitable resource distribution. In the SUS, some services have the EMR while other services, predominantly HBUs, are using paper-based referrals that require patients to hand deliver communications between physicians.

In the second theme, **Physician Accountability**, **f**ocus group participants acknowledged their own mistrust in HBU physicians, and a bias against the quality of HBU care delivery. Mistrust and lack of confidence often arises from inadequate communications between providers. In our study, participants identified physician education as one means to improve their communications with HBU physicians. Study participants desired orientation and ongoing education with respect to SUS functions and operations, particularly the referral process. Education could be designed in order to increase trust among professionals at the different levels of care, to avoid unnecessary referral, and not to forge “urgent” referrals only to gain access to the services.

Communications training has become a popular way to decrease communications failures within health care systems [19]. The majority of communications training, however, is at the team level [20,21] or within single healthcare organizations [22] where the predominant focus is on enhanced patient safety outcomes. Some literature describes services collaborative and networks, such as surgical services collaborative which have improved the quality and safety of service delivery through communications strategies that prevent communications breakdowns between providers [23]. Communications competencies for patient-physician and physician-to-physician communications are considered core competencies in many medical education programs [24]. Communications competencies encompass knowledge, skills and attitudes: transformation of attitudes, in particular, takes time. As noted by Frenk et al. [25], physicians play a crucial role in mediating the application of knowledge to improve the health of individuals and the community. Specialists’ negative attitudes towards primary care physicians may be a significant hurdle to overcome as the SUS strives to become an integrated health system. These biases must be overcome if specialists are going to increase communications and appropriately redirect patients to primary care services. One US study analyzed over 4000 physician self-reports of communication activities [12]. Physician-to-physician communications were enhanced by resource availability (e.g., staff support). Physicians tended to follow through less with colleagues in impoverished areas. Communications are particularly important in healthcare contexts where patients have multiple co-morbidities.. Traditional models of chronic care delivery rarely consider the practical realities of how clinicians, teams, and systems will work together [26].

Information or data volume can negatively influence physician communications [14] and the referral process. In an earlier study, Poon et al. [27] found that primary care physicians reviewed 1000 individual diagnostic studies weekly and spent an average of 74 min per day doing results reviews. Technology, such as electronic medical records (EMRs) is a strategy for information and knowledge management [10]. Technology, however, has its own challenges and workload demands [28].

As an example, the US Veterans Administration (VA), an extensive integrated health system, uses a sophisticated EMR to link providers across its system. In a study of one regional VA network, despite the EMR, providers reported problems with missed patient test results and subsequent treatment delays [29]. One identified EMR problem was physicians’ capacity to customize results notifications to control for volume overload—introducing human variability and error. The authors recommended standardized protocols across systems for better results management [30]. The presence of parallel referral systems is associated with systems fragmentation and communications breakdowns [29].

A review of seventeen e-referral systems from around the world (e.g., the UK, the US, Canada, Australia, Finland) found that these systems had varying degrees of success with respect to improved access to specialist care, reduced wait times, timeliness and quality of referral communications, accurate health information transfer, and service integration [31]. In every instance, success depended on engagement of key stakeholder groups and systems flexibility—to adapt to changing social-political conditions.

Our third theme was **Patient Accountability or Vulnerability**. In complex systems, patients have difficulty understanding and negotiating different service levels and referral processes—particularly when they are expected to be the ‘third party’ conveying critical information between services or providers. Although some study participants felt that patients abuse the SUS by bypassing the established referral process and seeking care from specialists or emergency services on their own, the majority of focus group participants described patients as vulnerable, naïve systems users. A study conducted in seven Brazilian hospitals found that the majority of patient respondents (77.1%) resorted to hospitals as their first choice of health facility because of “absence of professionals” in their communities [32]. Our study participants related similar accounts of patients telling them they were sent to the hospital because doctors were not available in their HBUs. Integrated health care systems have multiple entry points, and patient navigation, as mentioned by some of our focus group participants, is one approach for “improving the journey” for patients and providers [33]. In one Canadian province, cardiac navigators acted as a liaison between patients with cardiovascular problems and different services and providers: these navigators facilitated the delivery of timely information (e.g., tests) to enhance referrals. This particular navigation model also uses technology as a central repository for standardized information on service-specific referral requirements, and publicly accessible cardiovascular resources and contacts [34].

In Brazil, appropriate HBU use will depend on a stable supply of primary care physicians with sufficient government resources and supports to develop sustainable patient relationships and primary care-specialist relationships. Otherwise, patients will continue to mistrust HBUs and seek out specialist services on their own. Primary care physicians contribute to patient mistrust by over-referring patients to specialists—conveying a message (to patients and specialists) that they are not competent care providers. Ineffective communications between service providers accentuates specialist mistrust in HBUs. 

For the last theme, **Specialist Recommendations**, study participants made recommendations for systems change. Interestingly, no one identified the importance of physicians (HBU, specialists) being involved in the design of referral processes. They emphasized the role of government and health care administration in ensuring automated, accessible patient information for physicians at all systems levels. Successful health care innovation projects require physician champions and other stakeholder engagement to spread change within and across integrated health services [34,35].

Our study and other research reinforce the importance of government support for equitable resource distribution across the Brazilian SUS, particularly in HBUs that are a critical health service entry point for the public. As noted throughout the other themes, without sufficient resources, primary care physicians go elsewhere. Without access to effective primary care services, patients go elsewhere. Due to HBU resource deficiencies, specialists have mistrust and lack of confidence in primary care services. Problems with the referral process may be symptomatic of inequitable resource distribution, including sufficient numbers of well-informed, well-educated primary care providers.

Investment in technology is often considered a panacea for health care systems integration. We often focus on the technology and overlook how providers, as decision-makers, “create, validate, codify, share knowledge and make decisions” [36]. Strong working relationships among providers reinforce the knowledge process and lead to better results in complex health care systems. There are many challenges associated with the SUS that the government and health care administrators must address, but one of the most cost-effective solutions may be more education and engagement among primary care physicians and specialists to enhance referral communications at a relational level and instill trust and confidence in communications across service/systems levels [10]. As one participant said: “We need to both properly follow the patient. Our communications reflect our service to patients.”

## 5. Limitations

The results reported in this qualitative research reflect the experiences and perceptions of specialist resident physicians in Brazil: the young age of the sample and their ‘newness’ in the system may be one limitation. Other stakeholder perspectives (e.g., HBU physicians, patients, health care team members) need to be considered. At the time of the study, a computerized information system was under development in some settings and not in others. The implementation of this new technology was discussed by the focus groups, as promising (e.g., faster communications, better coordination) and frustrating (taking a long time to implement). Other variables mentioned in the focus groups (e.g., booking systems, organizational culture, public education, physician education) were superficially addressed in this study and need further investigation.

## 6. Conclusions

There are systemic problems within the SUS that need government and health care administration intervention. Lack of sufficient resources is a significant problem within HBUs that creates a domino effect throughout the SUS. Our study, however, has also uncovered some important practice applications for management. Managers, within HBUs and tertiary care settings, can facilitate physician exchange of referrals by developing routinized forms with critical information that physicians can promptly complete. Referral forms should include priority criteria. Referral process improvements will require key stakeholder buy-in, particularly physicians who can act as champions of change at different service levels. Managers have oversight for booking processes that prioritize patients based on need. Until the SUS is functioning smoothly, patients will bypass HBUs and the referral process and show up at hospitals and emergency rooms. In these instances, congestion can be managed by triage: nurses and/or residents should be assigned to triage roles for patients without referrals. Where EMRs exist between services, managers can monitor patient records for proper completion, transmission and receipt of patient referrals. Where EMRs do not exist, managers can facilitate the referral process by assigning staff to copy referrals and send them to appropriate services/physicians on behalf of patients. Dedicated staff can use FAX, emails or phone call follow-ups to ensure that critical information has been received. Although physicians may be professionally accountable for completing referrals, managers can augment the referral process by supporting follow-through of referral forms. Our study participants indicated that the government and health care administration needs to do more public education like the use of multimedia resources to more information about how the Unified Health System works. Management should consider how to use valuable time with patients (e.g., while they are waiting to be seen), to offer education via different routes (e.g., printed handouts, video displays); and to determine whether patients need other health care team services (e.g., nutrition counseling, social services, nurse care). A review of patient needs should be part of the triage or screening process: trained staff can contact and connect patients with ancillary health care services while they wait to see a physician. Ideally, physicians will develop their own strategies for engaging more with each other across services. Physicians, however, have heavy workloads, and they require the assistance of management to streamline the referral process across different levels of the SUS. A collaborative process is needed between management, physicians and other members of the health care team to create structures and processes that promote appropriate assessment, prioritization and referral of patients for care. Innovation work and regular communications by SUS management at different systems/service levels can model the way for physicians and the rest of the health care team.

## Figures and Tables

**Table 1:** Participants’ demographic profile.

Focus Group (FG)	Population	Sample		Age Average (Years)	Sex			
					Male		Female	
	n	n	%		n	%	n	%
FG1 Gynecology/Obstetrics	15	8	53.3	25.6	2	25.0	6	75.0
FG2 Pediatrics	20	17	85.0	26.8	4	23.5	13	76.5
FG3 Pediatrics	15	12	80.0	27.3	3	25.0	9	75.0
FG4 Surgery	11	7	63.6	26.0	4	57.1	3	42.9
FG5 Medical Clinic	31	10	32.2	28.0	4	40.0	6	60.0
TOTAL	92	54	58.7					

**Table 2:** Coding Schema.

**Theme: Government/Administrative Accountability**			
**Sub-Themes/Categories**			
**Physician workloads** Patient Volume Urgency/Priority Time Bureaucracy	**Physician turnover** No career Unstable physician-patient relationships	**Referral Process** Untrained staff Patient prioritization Triage/pre-screening Standardization No direct links	**Resources** Inequitable resource distribution
**Theme: Physician Accountability**			
**Sub-Themes/Categories**			
**HBU primary care physicians** Lack of education Forwarding therapy, passing the ball No follow-up/continuity Professional failure		**Specialists** Distrust in HBU Prejudice towards basic care No consideration, conversation	
**Theme: Patient Accountability or Vulnerability?**			
**Sub-Themes/Categories**			
**Accountability** De-valuing primary care Cultural phenomenon		**Vulnerability** Naivety No place to go/“city spawn” Unrealistic demands/“fragile third party”	
**Theme: Specialist Recommendations**			
**Sub-Themes/Categories**			
**Government/Administration** Automated, accessible records at all systems levels Public education Sufficient doctors	**Physicians** Professional accountability Regular communications		**Public/Patients** Ombuds service Education
